# Feeling at Home in the Wilderness: Environmental Conditions, Well-Being and Aesthetic Experience

**DOI:** 10.3389/fpsyg.2020.00402

**Published:** 2020-03-13

**Authors:** Helga Synnevåg Løvoll, Knut-Willy Sæther, Mark Graves

**Affiliations:** ^1^Department of Physical Education, Volda University College, Volda, Norway; ^2^Department of Religious Studies, Volda University College, Volda, Norway; ^3^Graduate School of Psychology, Fuller Theological Seminary, Pasadena, CA, United States

**Keywords:** aesthetic experience, awe, beauty, natural environment, sublime, well-being, wilderness, wonder

## Abstract

Environmental conditions affect one’s aesthetic experience in natural environments. Understanding that effect requires accounting for the conditions affecting one’s attention and experience. Rather than attempt to reduce and control environmental factors, we compare two similar groups during naturally occurring, intense and overwhelming conditions and examine the relationship between common characteristics as well as environment and group differences. Participants undertook a 5-day, winter, wilderness adventure training course designed to challenge their considerable wilderness and leadership skills under two different extreme weather conditions but within the same wilderness area (*n* = 47 full participation). In addition to pre- and post-adventure questionnaires, participants responded daily during the wilderness experience to briefly describe a self-selected, strong experience of nature; characterize its associated feeling states; and answer questions probing eight aesthetic aspects of the experience. Participant strong experience of nature related to hedonic and eudaimonic feelings in different ways depending upon environmental conditions. In particular, strong correlations occurred between agreement ratings with “I felt at home in nature” daily experience reports and satisfaction with life and personal growth trait measures, but primarily during sunny and cold conditions on a high plateau (PG: Pearson *r* = 0.51; SWL: *r* = 0.70) and not significantly in stormy and wet weather in a mountain forest. In addition, experience narratives that correspond to strongest agreement to feeling at home in nature were examined for shared themes and synthesized into six dimensions: focus on sensory experiences at a particular moment, self-reflection, wonder, appreciation of beauty, positive emotions, and insight of relation to nature. These findings actualize the notion of wonder, aroused by sudden feelings or by reflection, as a salient ingredient in feeling at home in wilderness. The finding of feeling at home in nature, as the most important feature relating to feelings and well-being, is discussed in relation to self-awareness, philosophical thinking, and potential ethical awareness.

## Introduction

When moving outdoors into the wilderness, various aesthetic experiences take place. In a sweeping landscape just moving one’s head may change one’s experience radically, so outdoor aesthetic experience is dynamic in a way that differs from looking at a piece of art (Chenoweth and Gobster, 1990, p. 2). In wilderness, the always shifting conditions over spatial perspectives and time result in multi-dimensional stimuli across all one’s senses. By selective interest, one might choose to pay attention to shifts of environmental conditions, focus on certain objects in wilderness, attend to inner processes of mental or spiritual states, or simply struggle for satisfying needs and comfort in harsh conditions. From moment to moment, one’s selective attention shifts among sensory stimuli and one’s felt experience of them. William James likens one’s attention to a “stream of thoughts,” and one’s selective interest plays a key role in understanding experience in contrast to utter chaos (James, 1890, p. 402). Wilderness adventure provides considerable potential for complex experiences, as there is room for silence, comfort, and contemplation on one hand, and challenging, even terrifying, surprising and overwhelming situations on the other. In this paper, we intend to identify core characteristics of the aesthetic wilderness experience in Norwegian winter mountains.

Studying the complex, equivocal, and perplexing phenomena of aesthetic experience and well-being in a wilderness context requires multiple perspectives including both holistic approaches–to ensure study of the actual phenomena in their long-standing historical context–and reductionist approaches to enable empirical discovery and analysis sufficient for novel insight into their enigmatic interrelationship. Although the need for such a span occurs regularly in the emerging empirical study of many complex phenomena, the examination of aesthetic experience in particular demands attention to that complexity, as the holistic-reductionist spectrum can itself be an essential aspect of the aesthetic experience.

Aesthetic experiences are important for humans as they affect mood and indirectly promote well-being (Mastandrea et al., 2019). Our approach to aesthetic experiences in nature is in line with Tomlin (2008) reflections that stress the transformative and evaluative dimensions of aesthetic experience (rather than only its analytical or defining characteristics). For Tomlin, an experience of high value gives the subject a new sort of consciousness not accessible through other experiences. “What transforms [a] kind of perception to an aesthetic experience is that it becomes an ‘event’.” (Tomlin, 2008, p. 7). In the process of understanding experiences to be transformative and evaluative, there are qualitative differences among several facets of the experience, and among these: the role of beauty and sublime dimensions in aesthetic experiences (different characteristics), the role of hedonic and eudaimonic experiences in well-being (different affective dimensions), and the role of stimulus-driven and goal-directed attention in the way one orients in wilderness (different involuntary and voluntary attention).

Environmental aesthetics identities that, because aesthetics depends upon attention and its evaluative and transformative effects, it also depends upon *to what* one attends. The aesthetic experience is an experience of a particular time and place, i.e., “event.” There are commonalities among aesthetic experiences that enable them to be collected as “aesthetic experience,” but one does not have a general experience aesthetically, one only has particular aesthetic experiences, and thus the environmental context plays a crucial role. The field of environmental aesthetics spans many approaches to how one appreciates nature (Carlson, 1998, 2000), and one crucial topic in environmental aesthetics is the relationship between aesthetic experience and aesthetic judgment (Stecker, 2005). For Stecker, these two are strongly intertwined, and for him the aesthetic experience is not only about what is pleasing, but about what one values as important. Such an approach opens up a complex understanding of what is happening in an aesthetic experience. According to Berleant (1998), environmental aesthetics needs to cover more than what is, in one sense, visual pleasing. The experience of nature, or the surrounding environment, is about several aspects, such as space, volume, time, movement, color, light, smell, sound, touch, order, and meaning. For Berleant, in environmental aesthetics the experience of beauty in nature has to be understood in a complex way as “… the pervasive aesthetic value of an environmental situation” (Berleant, 1998, p.118).

Related to aesthetic experiences in nature is the notion of wonder. Wonder is undoubtedly a complex phenomenon. One way to understand the relationship between aesthetic experiences in nature (such as beauty and the sublime) and wonder is that the former activates the latter. The experience of beauty stimulates wonder: What is this beauty I am experiencing? Why is it so? We might reflect in a similar way concerning the sublime (Sæther, 2017). Robert Fuller relates these phenomena by saying: “Wonder most frequently occurs as a response to something that strikes us as intensely powerful, real, or beautiful” (Fuller, 2012, p. 70). In one sense, as one encounters nature, one might talk about the experience of wonder, in line with experience of the sublime and beauty. However, wonder is a larger, more overreaching, profound, and subtle concept (Fuller, 2012).

Wonder has at least two dimensions: as something in nature which evokes a feeling of wonder and something motivating humans for reflection and further search for insight (Sæther, 2017). This motivation is a kind of an inner flow on the part of, for example, the scientist as described by Ralph Waldo Emerson: “Men love to wonder, and this is the seed of science.” (Ledley, 2009, p. 246). We can describe this inner flow as a shared experience for everyone in one’s search for understanding of the world. For Sophia Vasalou, such a flow is not only intellectual, as in a search for understanding, but also functions as a motivation for practices (Vasalou, 2012). Hence, in understanding wonder, this phenomenon can motivate toward an ethical awareness.

Both dimensions of wonder, those induced by sudden feelings and those motivated by reflection, can be explored as experiences with relevance for ethical awareness. An ethical awareness is important to feel deeply connected to nature as one of the most important virtues of our time, addressing how to respond recording to environmental crisis (Vetlesen, 2015). For Seel (1998) our aesthetic experiences in nature are pointing toward an ethical dimension of what we strive for and hope for: “… the aesthetic of nature is […] simultaneously part of an ethics of the individual conduct of life […] for aesthetics, being concerned with specific forms of and opportunities for process-oriented activity, is generally part of an ethics of the good life.” (Seel, 1998, p. 342).

Another, although closely related, way of relating aesthetics and ethical awareness is developed by Bergmann. He coined the term aesth/ethics to emphasize aesthetics as strongly intertwined with ethics (Bergmann, 2011). Bergmann shows that aesth/ethics, with the slash, indicates that ethics is embedded continuously in perception. If ethics is defined as a discursive reflection on moral problems, we cannot exclude people’s mental capacities and separate aesthetic competence from moral competence, thus the perception of moral problems must be prior to their reflection and possible solution, he says (Bergmann, 2011). Bergmann’s concept is to develop a specific contribution to eco-theology, and he does so by exploring three concepts, with relevance for our findings: inhabitation, *Beheimatung*, and atmosphere.

The first, inhabitation, recalls us to take seriously the perception of space and life, and works as a first step for the following two. “Beheimatung,” the German word for making oneself at home, addresses the question of belonging or feeling at home. When addressing “feeling at home in nature,” we need to have in mind that it might mean different things in various contexts. For Bergmann, the question one needs to ask is how to make oneself at home at “Earth, our home” which we collectively are spoiling. The “feeling at home in nature” is an experience or feeling taking place in the extension of aesthetic experiences in nature. For Bergmann, aesthetic experiences is also about a self-aware human reflection on one’s living-in-particular-surroundings (Bergmann, 2006, p. 336). In addition, Bergmann’s notion of atmosphere is relevant for our context, because it emphasizes the interconnectedness of the inner and outer, the bodily and the spiritual, the surrounding and the inhabitation. An awareness of who we are, and how we are interconnected with nature is of major importance. A lack of awareness creates alienation and awareness is a skill to be nourished and developed (Bergmann and Eaton, 2011, p. 3). For Bergmann and Eaton, awareness is an aspect of *how* we sense and perceive the world in a specific way. The way of seeing things is prior to the way of acting, it is about our senses and perceptions and how we actually pay attention. It includes all our senses: what we see, taste, hear, and touch. Such an aesthetic awareness influences the kinds of questions we ask, how and what we reflect upon, and ultimately how we answer our queries (Bergmann and Eaton, 2011, p. 3). Aesthetics, as the way of seeing things, is according to Bergmann a trajectory to ethical awareness.

The traditional understanding of beauty in nature relates to the pleasing dimension in experience specific objects (beauty in small scale) and the pleasing of beautiful scenery (beauty in large scale). The former is the heritage from Kant, the latter the tradition from Joseph Addison and Francis Hutcheson (Sæther, 2017). Compared to the experience of beauty we might describe the experience of the sublime as more holistic and evoking a wide range of feelings (Graves et al., 2020). John Baille argued for this back in 1747. He says the sublime is a function of the grandeur of objects (in nature), while the experience of beauty takes place in a smaller scale. Further, awe is traditionally understood as a response to the sublime. Fred D. Ledley describes the sublime as causing a sense of exaltations and awe. William Wordsworth describes such experiences as “impressions of power, feeling of apprehension, dread, fear, or wonder” (Ledley, 2009, p. 248). The sublime also includes a sense of duration in which “individuality is lost in the general sense of duration belonging to the earth itself” (Ledley, 2009). Alexander (2014, p. 52) expresses the sublime as “a pleasure in the way that nature’s capacity to overwhelm our powers of perception and imagination is contained by and fuels our rational comprehension.” In the extension of both the experience of beauty and the sublime, the experience of wonder is relevant to emphasize. The experiences of beauty and the sublime are intertwined with the experience of wonder. Wonder is evoked by a surprising situation, such as changes in weather, having a novel perception of something in nature.

We understand the sublime as “… as a pleasure in the way that nature’s capacity to overwhelm our powers of perception and imagination is contained by and fuels our rational comprehension…” (Alexander, 2014, p. 59), and the sublime evokes “a sense of exaltation and awe, a sense of duration in which individuality is lost…” (Ledley, 2009, p. 248). To examine the sublime empirically, we draw upon an emerging literature on the psychology of awe, where Keltner and Haidt (2003) finds beauty the most predominant experiential theme, and Yaden et al. (2018) finds natural scenery the most predominant trigger eliciting awe. Although awe may include constructs unrelated to the sublime and the sublime has other aspects in addition to awe (Sæther, 2017), considering awe in an aesthetic context enables bridging empirical investigations of awe and aesthetics to begin creating an empirical foundation for studying experiences of the sublime. Considering levels of connectedness-to-nature as traits, the intensity of transcendent and awe-inspired experiences seems to increase (Davis and Gatersleben, 2013). However, in this latter study, connectedness-to-nature appeared to be a trait that can be trained.

Investigating traits for aesthetic nature experiences, a two-factor structure was recently identified: One relating to traditional perceptions of beauty, typically focusing on beautiful scenery, while the other relates to the sublime, typically by deeper immersion and experiences of awe (Graves et al., 2020). In the beauty-dimension (seven items), this correlated with strong relationships and communion with nature, while in the sublime-dimension (eight items), this correlated with the importance of fulfillment and peace. In the current study, we explore if sublime aspects of aesthetic nature experiences also yield *situational* wilderness experiences, and if so, how we can characterize and understand these experiences. Nature has an important impact of our overall well-being. From an environmental psychology perspective, the presence of nature is first and foremost known as having a stress reducing effect (see for example Hartig et al., 1991; Laumann et al., 2003). To be in natural environments is an effective arena for emotion regulation and important for one’s everyday well-being (Johnsen and Rydstedt, 2013). Thus, daily hikes help to reduce stress and regulate emotions. Sensory experiences in nature are moreover a source of positive emotions (Ballew and Omoto, 2018) and being more vitalized (Ryan et al., 2010). In the outdoor setting, one’s social relations also improve, in being more caring for each other (Weinstein et al., 2009).

Moreover, certain activities in the natural environment feed different positive emotions: While pleasant feelings associate with life satisfaction, striving to use one’s potentials or seeking meaning relates to eudaimonic well-being (Vittersø et al., 2010; Vittersø and Søholt, 2011; Vittersø, 2016). Both dimensions are important in a fully functional life. Wilderness thus carries potential for complex aspects of well-being, where aesthetic *pleasure* might associate with life satisfaction and aesthetic *interest* might associate with personal growth. Awe, as a positive emotion taking place in aesthetic nature experience, transforms us toward a reorientation of our lives, goals, and values (Fuller, 2012; Sæther, 2017). While the function of emotions to some degree is known in well-being research (Vittersø, 2016), the identification of intentionality: when and why these emotions occur in the natural environment, are less known. Both aesthetic pleasure and aesthetic interest are identified during wilderness experiences, whereas aesthetic interest most typically can be interpreted to the active approach to valuing the natural environments as sacred, construction of new meaning, and feeling a connection with the powerful unseen forces of wild nature (McDonald et al., 2009), which could correspond to understanding the sublime.

The distinction between the influence of aesthetic nature experiences and the mere presence of nature is hard to draw, as our attention shifts between paying attention to sensory stimuli and intentionality, by selective interest. In the brain, there appears to be at least two systems connected to attention: the role of the attention shift by the *orienting network* and the role of focusing attention by the *alerting network*. In addition, an executive network makes the overlap and selection between the different systems (Posner, 2008). These different attention systems are connected to the stimulus driven attention and the goal directed attention (Corbetta and Shulman, 2002). Although both systems interact in a situation of normal sensory experience, there is a selection process switching from one system to the other, called the “attention shift” (Broadbent, 1956), which is dependent on the competition of the different system processing network. This insight can be related to aesthetic nature experiences as these are embedded in perception, which according to Bergmann and Eaton is about seeing in a specific way, i.e., awareness and to pay attention (Bergmann and Eaton, 2011).

In the wilderness, it is likely that both the orienting and alerting networks provide aesthetic experiences in visual, hearing, smelling, tasting, and touching sensory experiences – both as basic qualities of nature and as sudden shifts or movements, what identifies as involuntary attention called “soft fascination” (Kaplan and Kaplan, 1995). Moreover, there could be more goal-directed attention in seeking for some special qualities, for example in looking for rare plants or animals or in striving to use skills to keep warm and dry, or even in striving to achieve a state of well-being, for example in being mindful. During a winter day in the snow, with skis and backpack, one may have all kinds of emotional experience, shifting from one moment to another. However, some experiences might be more important, as we pay attention to some aspects of the adventure when thinking of strong experiences of nature. Intense experiences in nature moreover carries the potential for rare, life changing experiences in the deep interpretation of peak-experiences (Maslow, 1976). The intense environmental context for the studies necessarily becomes a factor in the investigation enabling study of the relationship between environment and aesthetic experience of nature.

Outdoor environment provides many health promoting ingredients. For example, coastal landscape provides therapeutic values in experiencing emotional, embodied and often shared connections with the coast (Bell et al., 2015). There might be differences in personality traits and preference of places, whereas introverts prefer mountains more than extroverts, and introverts are happier in wooded landscapes than in open areas (Oishi et al., 2015). Emotional experiences of landscapes were moreover enhancing the relationship between place-identity and well-being in Swedish mountains (Knez and Eliasson, 2017). The more bonded one felt with the place, the better well-being. Taking these differences account, there might be differences in how the forest adventure differs from mountain adventure in understanding aesthetic nature experiences.

In mountain areas, there are many weather changes that could influence the overall judgment of the experience. Experiencing cold and wet circumstances could result in low scores on hedonic well-being but these experiences could be less relevant to eudaimonic well-being. Strong winds, heavy rain or snow or extremely cold could moreover influence our judgments and what we choose to attend to. As we tend to remember events based on experiences of peak, ends and specific emotions in our overall judgment (Fredrickson, 2000), weather issues might influence our perceptions of peak, ends and specific emotions, or they are less relevant. We assume that weather issues are important in the overall experiences of the wilderness adventure, but we do not know to what degree weather issues influence aesthetic nature experiences.

Our aim is to explore how the dimensions of aesthetics and well-being characterize *situational* experiences during 5-day, winter, wilderness adventures. Based on data collection from two, similar yet distinct, environmental conditions, we wish to identify structures that are common and divergent through these adventures. In addition, we found feeling at home in nature to be a very important dimension for aesthetics and well-being in our study, and we investigate that further.

We explore three complementary questions that relate aesthetic experience and well-being within a wilderness environment:

1. What characterizes core aesthetic dimensions in wilderness?
2. How do those aesthetic experiences affect well-being?
3. What is the role of aesthetics and its affective aspects associated with feeling at home in nature?

We do this by analyzing the core aesthetic dimensions and well-being measures using quantitative methods, identifying the significance of feeling at home in nature, and explore the aesthetic and affective dimensions of belonging predominantly using qualitative methods.

## Materials And METHODS

The holistic-reductionist methodological complexity needed for studying aesthetics and well-being in a natural environment affects at least two aspects of the present study. First, our empirical approach uses mixed methods with quantitative analysis of survey data and experience reports as well as qualitative text analysis of experience report narratives. Second, our theoretical approach focuses narrowly on how the aesthetic experience in wilderness adventure affects well-being and also branches out philosophically to include not only the narrow experience of beauty in nature but also the sublime, its associated feelings of awe and wonder, and feelings of interconnectedness, communion, and belonging in the wilderness experience. This multifaceted approach enables careful investigation of the full aesthetic experience and its interrelationship with a richer conception of well-being.

We examine one’s aesthetic communion with nature and strong experiences in an expansive and attention-demanding wilderness setting, in particular its effect on overall well-being. Toward that end, we examined students undergoing training to guide wilderness expeditions before, during, and after a 5-day, intense wilderness adventure designed to challenge their considerable wilderness and leadership skills. The students do not yet have skills that would be considered as expert or exemplary as leaders. Examining a participant pool with greater outdoor adventure skills than a typical adult, hopefully yields insight into the same phenomena experienced by numerous people during shorter and/or less intense wilderness adventures.

### Mixed Methods

A combination of quantitative measures and qualitative narratives were used to detect and explore details in the aesthetic experience of nature. Moments of subjective experiences identified by each participant as “strong experiences of nature” were narratively described and quantified using aesthetic and affective measures for the intensity of aesthetic dimensions and feelings. Using the participant’s rating of intensity for the experiences and feelings, corresponding narratives were selected to analyze using qualitative methods, to expand the research paradigm not only to include close-ended, but also open-ended data (Merriam and Tisdell, 2016). An iterative process was used in classifying the selected, high-intensity, experience reports, first investigating the total sample, and second, investigating similarities and differences between the two different contexts. Third, as quantitative methods identified feeling at home as a significant experience, thematic analysis was used to characterize feeling at home in the wilderness. By focusing on the daily moment chosen by participants as a strong experience and using the quantitative aspects of the experience reports to select narratives for qualitative analysis, mixed methods are well integrated (Teddlie and Tashakkori, 2009). In addition, questionnaires regarding aesthetic traits and well-being were conducted before and after each expedition, described below.

### Participants and Environmental Research Contexts

Because of the key role environmental context plays in *in situ* study of aesthetics, we describe a study with identical experimental design and similar participants undertaken in two different environmental contexts. The analytical methods remain the same between both studies, and because (pre- and post-adventure) trait instruments taken in a controlled (classroom) environment showed no differences between the participant groups, we can relate differences between groups to the environmental context.

#### Total Sample

Within the context of a formal education program in leading extreme outdoor wilderness adventures, a group of students from a University College in Norway were followed through their 5-day expedition to the winter mountains in 2017 in Norway (*n* = 26, *M*_age_ = 26.1 years, 42.3% females). 24 students reported on the pre- and post-tests, before and after the wilderness experience (92.3%), while 21 volunteered to answer questions during trip (81%). The following year, an additional study was conducted with a new group of students from the same University College in the same mountain area. From a group of 43 students (*M*_age_ = 24.5 years, *SD* = 2.6, 51.2% females), 37 students reported on the pre- and post-tests, before and after the wilderness experience (86%), while 26 students volunteered to answer questionnaires during trip (60% participation). Altogether 62 students out of 67 reported on pre- and post-measures of the wilderness expedition. Forty-seven of 67 reported on experience reports.

#### Environmental Context 1: Forest-Stormy

During the first wilderness expedition, there was a full storm for 4 days, and it was necessary for safety reasons to discontinue the plan to reach the high-altitude plateau over the tree line. As a consequence, the whole expedition took place in the forest. The student group was divided in three smaller independent groups, with three leaders (one female leader). They all moved in the same area, but in separate camps, not in sight of each other. Students lived in self-built snow caves some of the days. For simplicity we call this group “forest-stormy.”

#### Environmental Context 2: Plateau-Cold

During this wilderness expedition, it was sunny, but the temperature was very low (minus 25°C). This time, the groups succeeded in reaching the high-altitude plateau, and moved for a longer distance in the mountain. They lived in self-built snow caves some of the days. The group of 43 students were divided into four groups, each group with a leader (one female leader). The groups moved in a wide area, not in sight of each other. For simplicity we call this group “plateau-cold.”

### Instruments for Psychological Traits

Students were given (pre- and post-adventure) trait instruments in a classroom environment, including measures of satisfaction with life and personal growth.

#### Satisfaction With Life Scale

Five items for Satisfaction With Life Scale (SWLS; Pavot and Diener, 1993) were used on a Likert scale from 1 to 7 (e.g., “In most ways my life is close to my ideal,” “I am satisfied with my life”).

#### Personal Growth

We used a personal growth composite based on four different dimensions, each measured by three items: Curiosity (Amabile et al., 1994) (e.g., I enjoy to deal with new tasks presented for me), Absorption (Kashdan et al., 2004) (e.g., When I participate in an activity, I have a tendency to be so involved that I “forget time”), Complexity (e.g., I like to hear about new ideas), and Competence (e.g., I like to meet challenging tasks). Complexity and Competence were based on California Psychological Inventory (CPI) from International Personality Item Pool (IPIP, 2002; HPI Science ability HIC). Each item was measured on a bipolar scale from 1 to 5, ranging from “disagree” to “fully agree.” This measure of personal growth is used in other publications (Kopperud and Vittersø, 2008; Straume and Vittersø, 2015).

### Instruments During Nature Experiences

During the wilderness experience, participants were asked to record questionnaire responses in a hand-written diary.

#### Strong Experiences of Nature

Inspired by the Day Reconstruction Method (DRM; Kahneman et al., 2004) and Event Reconstruction Method (ERM; Grube et al., 2008), each student was asked to describe a strong experience of nature daily in a diary made especially for the data collection. The instruction was: “Thinking about this day in the mountains, select one event when you felt a strong experience of nature. Describe this strong experience.” Five lines with open space were available for the answer. Based on this strong experience, we asked students to report quantitatively on questionnaires regarding aesthetical nature experiences and feeling states. This diary was distributed at the start of the wilderness expedition and collected at the end of the expedition. Students were asked to report on the same questions for 5 days.

#### Feeling States

In relation to the self-selected episode, students were asked to report how intensely they felt during the episode. Three hedonic feelings (satisfaction, pleasure, and happiness) and three eudaimonic feelings (interest, engagement, and enthusiasm) were gauged on Likert scales (1–7) based on an adjusted version of the Basic Emotion State Scale (Vittersø et al., 2005).

#### Aesthetic Situational Nature Experiences

As we were unaware of a suitable questionnaire to examine aesthetic experience in nature, we tested eight novel aesthetic theory-generated questions: (1) I experienced beautiful scenery, (2) I was aware of small details in nature, (3) I appreciated variety in nature, (4) I felt everything was connected in nature (or: I felt everything in nature was connected), (5) I felt at home in nature, (6) I felt nature evoked wonder, (7) I felt beauty in nature evoked wonder, and (8) I felt nature evoked awe and respect. These questions do not operate as a scale to measure one phenomenon but give an opportunity to find empirical evidence to theory-driven questions capturing different dimensions of aesthetics. Each item was asked in Norwegian translation (the native language of participants) and measured on a Likert scale 1–7. To ensure similar meaning between English and Norwegian, the questions were based upon aesthetics literature available in English but formulated in Norwegian. The Norwegian questions were back translated into English and from this translation a new translation into Norwegian by an independent Norwegian-English speaking researcher was made.

### Human Subjects Review

Students were invited to participate during a regularly scheduled, wilderness leadership course. They were informed in writing that they could withdraw from the project at any time and for no reason. The data would then be deleted. Data was collected by paper and pencil, with no personally identifiable information, as only a participant-generated id was used. Students were asked to use an anonymous code (not identifiable for the researchers) to group the questionnaires together. Following national rules of personal information safety by the Norwegian Centre for Research Data (NSD), no additional written consent was necessary, given the process used to collect and store data. The project also was reviewed for appropriate informed consent though the university college where the study took place.

### Design and Procedures

Pre- and post-questionnaires of traits were distributed in ordinary classes the week before and the week after the wilderness expedition. As these classes were obligatory to join the wilderness expedition, all students had the opportunity to participate in the survey.

During each expedition, students reported daily on their experiences in a diary, collected afterward by the researcher and their research assistants, who were also wilderness leaders. Students were friendly reminded in the evenings to fill out their daily experiences after they had come safely into their sleeping bags in the evenings, using head torch and pencil to fill out the questions.

### Quantitative Analysis

#### Descriptives

Collapsed measures of the 5 days experience sampling on each question of the aesthetic situational nature experience were calculated to find mean scores and standard deviations for the wilderness experience at group level (see Table 1). Three analyses were performed: the entire sample and the split forest-stormy and the plateau-cold samples. Analyses were performed in SPSS, version 25.

**TABLE 1:** Means for situational aesthetic nature experiences.

**Aesthetic situational nature experiences**	**Forest-stormy Valid (*n* = 12)**		**Plateau-cold Valid (*n* = 20)**	
	***M***	***SD***	***M***	***SD***
*I experienced beautiful scenery*	4.63	1.18	5.45	0.87
*I was aware of small details in nature*	5.17	0.92	5.19	0.94
*I appreciated variety in nature*	5.45	1.12	5.47	0.96
*I felt everything was connected in nature*	4.79	0.81	4.77	0.94
*I felt at home in nature*	4.89	1.06	5.14	1.03
*I felt nature evoked wonder*	4.81	1.49	4.92	1.19
*I felt beauty in nature evoked wonder*	4.33	1.60	4.67	1.25
*I felt nature evoked awe and respect*	5.05	0.92	4.87	1.58

*Only respondents with no missing were included.*

#### Correlations

Hedonic and eudaimonic feelings were correlated with eight different items of the aesthetic nature experience. Satisfaction with life and personal growth from the post-measure of the wilderness expedition were correlated with “felt at home in nature.”

### Qualitative Analysis

The coding from subthemes (events) and main themes (context) toward synthetic dimensions was discussed and agreed upon by two investigators. Experience reports were selected for closer investigation when participants also answered the question of “felt at home in nature” with the strongest Likert response. Only quotes that were also reported as 7 on the Likert Scale 1–7 were included. With this selection, 13 quotes from the forest-stormy-sample, and 26 quotes from the plateau-cold-sample satisfied the selection criteria, suitable for narrative investigations. First, all narratives were read through and coded by two independent researchers. Next, dimensions across the narratives were agreed upon, based on thematic understanding of the narratives. Third, the researchers analyzed each narrative in terms of synthetic dimensions, which were deduced based on shared themes in the narratives. The inductive analysis process follows the strategy of theme-oriented analysis (Braun and Clarke, 2006).

## Results

### Descriptives

Mean scores for each of the items from the questions of aesthetic situational nature experiences indicated similarities and differences on group level between the forest-stormy- and the plateau-cold-sample. Calculated with the two-sample *t*-test, the first question “I experienced beautiful scenery” was reported as stronger in plateau-cold than in forest-stormy. This finding was close to significant (*p* = 0.051). Changes in other scores were not significant between these small samples (see Table 1 for more details). All data was checked to be normally distributed.

### Feeling States

For hedonic feelings, reliability tests showed a Cronbach’s alpha α = 0.80 (in forest-stormy) and α = 0.96 (in plateau-cold). For eudaimonic feelings, α = 0.95 (in forest-stormy) and α = 0.95 (in plateau-cold).

### Correlations

In the hedonic and eudaimonic measures of how the strong experience of nature felt, there were similarities between the two environmental conditions on some items, but others differed in their correlation to aesthetic questions. For example, “beauty in nature evoked wonder” felt more hedonic in forest-stormy while more eudaimonic in plateau-cold. To be more aware of details in nature (item 2), felt more hedonic in forest-stormy and more eudaimonic in plateau-cold. The most affective dimension of the aesthetic nature experience related to “felt at home in nature” (item 5), which had the highest correlations with both hedonic and eudaimonic feelings for the whole sample. During plateau-cold, this item correlated highly with both hedonic feelings (Pearson *r* = 0.82, *p* < 0.001) and eudaimonic feelings (*r* = 0.91, *p* < 0.001), while in forest-stormy, this dimension correlated with hedonic feelings (*r* = 0.63, *p* = 0.011) but not eudaimonic feelings (*r* = 0.22, *p* = 0.459) (see details in Table 2).

**TABLE 2:** Correlations between aesthetic nature experiences and feeling states.

	**Total sample**		**Forest-stormy**		**Plateau-cold**	
	***Hedonic***	***Eudaimonic***	***Hedonic***	***Eudaimonic***	***Hedonic***	***Eudaimonic***
I experienced beautiful scenery	0.58*	0.40*	0.68**	0.59*	0.54*	0.46*
I was aware small details in nature	0.42*	0.55**	0.60*	0.45	0.33	0.60*
I appreciated variety in nature	0.45*	0.53**	0.55*	0.48	0.40*	0.61*
I felt everything was connected in nature	0.29	0.40*	0.75**	0.41	0.45*	0.68*
I felt at home in nature	0.75**	0.61**	0.63*	0.22	0.82**	0.91**
I felt nature evokes wonder	0.43**	0.43*	0.42	0.27	0.44*	0.59**
I felt beauty in nature evoked wonder	0.48**	0.51**	0.64*	0.48	0.38	0.60*
I felt nature evoked awe and respect	0.27	0.35	−0.05	−0.5	0.37	0.46*

*p < 0.05, **p < 0.01.

Five items had higher correlations with hedonic feelings during forest-stormy than plateau-cold, but these differences did not reach the level of significance. Similarly, seven of the items had higher correlations with eudaimonic feelings during plateau-cold than forest-stormy. “Felt at home in nature” reached significance (*z* = 3.16, *p* < 0.01), as did “felt nature evoked awe” (*z* = 2.54, *p* < 0.05).

Hedonic and eudaimonic feelings were related to well-being in different ways, too. In the whole sample, hedonic feelings correlated with personal growth (*r* = 0.42, *p* = 0.015). Looking closer to forest-stormy, hedonic feelings were important for personal growth, but did not reach a level of significance (*r* = 0.38, *p* = 0.184). Eudaimonic feelings were also relevant for personal growth (*r* = 0.34, *p* = 0.260). Satisfaction with life was not related to either hedonic (*r* = 0.04, *p* = 0.896) nor eudaimonic feelings (*r* = −0.13, *p* = 0.677). In plateau-cold, hedonic feelings correlated with both satisfaction with life (*r* = 0.47, *p* = 0.033) and personal growth (*r* = 0.51, *p* = 0.022). Eudaimonic feelings did not reach levels of significance for correlation with satisfaction with life (*r* = 0.28, *p* = 0.229) but correlated with personal growth (*r* = 0.49, *p* = 0.033).

The question of feeling at home in nature from the experience reports was correlated with life satisfaction and personal growth. In forest-stormy, personal growth correlated with feeling at home, approaching levels of significance (*r* = 0.49, *p* = 0.073). In plateau-cold, feeling at home in nature correlated with both life satisfaction (*r* = 0.70, *p* < 0.001) and personal growth (*r* = 0.51, *p* = 0.022).

### Regression Analysis

In the combined sample, a regression analysis of the *in situ* aesthetic nature experiences for all eight items were used as the independent variables of the post-measure of personal growth. This model had an explained variance of *R*^2^ = 42.4, *p* = 0.151. Only item 8 “I felt nature evoked awe and respect” was significant in the model, as a negative predictor (*t* = -2.4, *p* = 0.03). A second model was tested based only upon the almost significant item 5 “felt at home in nature” as the independent variable, which explained less variance, *R*^2^ = 13.1, *p* = 0.04, but in this model, “felt at home in nature” was a significant predictor (*t* = 2.2, *p* = 0.04).

### Qualitative Analysis

Narratives that corresponded to “felt at home in nature” (= 7 on the Likert scale) were extracted from SPSS and copied into a word file. Next, the first and second author read independently through the material and some common characteristics appeared: looking at phenomena of light, such as stars, aurora borealis, the sun, and campfire. Participants also frequently observed phenomena related to fauna and flora, such as trees and traces of animals. Many report experiences of silence in nature. Some narratives also report of some sort of “movement” as strong experience. This movement is either when they are moving through a particular environment, e.g., the forest, or a movement from one local environment to another, expressing a transition, e.g., moving from the forest into the high mountain. Further, the narratives were related to some broader synthetic dimensions after a full theme-oriented analysis. See an extraction of this analysis in Table 3 (full data available in Appendix 1).

**TABLE 3:** Example of the analysis process and developing sub-, main-, and synthetic dimensions.

**Environmental context**	**Narratives**	**Sub-themes (event)**	**Main themes (context)**	**Synthetic dimensions**
Forest-stormy	Come through the pine forest and feel the sun warming in the face and the sound of the wind whizzing in the trees	Sun warming, sound, forest	Sounds of wilderness	Description of a certain moment with focus on sensory experiences
Plateau-cold	When all the lights from the headlamp were extinguished and the stars appeared	Stars. Light	Contrast darkness/light	
	Look at the stars with a friend while we melted snow in 20 minus	Stars. Light. Cold		
Forest-stormy	The feeling of being alone in the pine forest	Being alone, forest	Forest reflection/alone	Description of self-reflection
	Met on some fresh animal tracks	Animal tracks	Forest, surprise	Description of wonder
Plateau-cold	Look at animal tracks in the forest			
	Saw many animal tracks			
	Ice-covered bench	Ice fascination	Fascination of details, surprise	
	Blackcock (big bird) that flew up	Bird		
Forest-stormy	Snow cave. How amazing is it that you can build something so nice, cozy and warm by snow? Totally insanely nice and an aha experience	Snow cave	New snow experience	Description of a certain moment of appreciation of beauty
	We walked past a lovely old pine tree. The branches and trunk twisted around in a stylish way	Tree, forest	Fascination and aesthetic judgment of details/forest	
Plateau-cold	When we walked between pine and birch and sang “In the forest I am free”	Walking and singing. Forest	Enjoyment	Situations focusing positive emotion
	When I had time to enjoy breakfast with the morning sun in the middle of me	Warmth from sun. Light	Pleasure	
	Walk through the woods of skiing with everything you need to survive on your back makes you feel strongly connected to nature	Connection to nature, moving, carrying backpack, forest	Reflection on relation to nature when moving	Description of a certain moment with focus on sensory experiences and insight of relation to nature

Narratives analyzed by synthetic dimensions were: (1) Description of a certain moment with focus on sensory experiences: e.g., “To come through the pine forest and feel the sun warming in the face and the sound of the wind whizzing in the trees”; (2) Description of self-reflection: e.g., “The feeling of being alone in the pine forest”; (3) Description of wonder: e.g., “Met on some fresh animal tracks”; (4) Description of a certain moment of appreciation of beauty: e.g., “We walked past a lovely old pine tree. The branches and trunk twisted around in a stylish way”; (5) Situations focusing positive emotions, e.g., “When I had time to enjoy breakfast with the morning sun in the middle of me”; and (6) Insight of relation to nature: e.g., “Walk through the woods of skiing, with everything you need to survive on your back makes you feel strongly connected to nature.” Some of the narratives included more than one synthetic dimension among these six synthetic dimensions.

## Discussion

Strong experiences of nature inform our well-being in complex ways. In wilderness, strong experiences of nature relate to personal growth, and sometimes also satisfaction with life. “To feel at home in nature” arises as the most important feature relating to aesthetic nature experience. This feature includes sensory experiences as well as reflections, which actualize the notion of wonder. In order to understand aesthetic nature experiences in the wilderness, the study of winter expeditions yields some clarity into the effect of environment on aesthetic experience and well-being as well as opportunities for theoretical insights. There appear to be both some stable elements and some context-dependent elements within the aesthetic nature experience that require careful analysis.

### Core Characteristics of Aesthetic Experiences in the Wilderness

When interpreting the results from the combined winter expeditions, the eight questions regarding aesthetic nature experience account for 42.4% of the variance of personal growth. Although the regression analysis in the small sample did not reach the level of significance, aesthetic nature experiences seem to be powerful in understanding this aspect of well-being. However, the items seem to vary, and dividing participants into their two different environmental contexts thus informs about similarities and differences in some of the items. In order to understand more of the sublime dimension, which we strongly identify with personal growth (Graves et al., 2020), a deeper approach is necessary.

In both contexts, strong experiences of nature included same levels of “awareness of small details in nature” and “appreciations of variety in nature,” indicating that these dimensions were equally important across the different contexts. The groups differed in intensity of experiencing beautiful scenery, which in forest-stormy was different due to wind and snow. On other typical qualities, there were some slight differences, such that “felt at home in nature” was more intense during the plateau-cold sample. Also, both questions of wonder were more intense during the plateau-cold sample. On the other hand, awe and respect were stronger during the first, forest-stormy year. Although these differences were informative, the sample sizes (of participants who rated belonging Likert = 7) were too small for the differences to reach significance.

Affectively, there were also some similarities and differences in the two different winter expeditions. All eight aesthetic items relate to positive emotions, but in different ways. For example, while the feeling that everything was connected in nature and feeling at home was hedonic during forest-stormy, it turns out to be more eudaimonic during plateau-cold. One explanation could be that during forest-stormy, students had to work hard in order to keep warm and dry, and when they succeeded in this, they felt connected to nature, which is a hedonic feeling. During plateau-cold, focus was much larger than keeping warm and dry, as during this expedition they skied for a long distance and could feel connected to nature in more complex ways, both hedonic and eudaimonic. During plateau-cold, “felt at home in nature” felt more eudaimonic than during forest-stormy. During forest-stormy, “felt nature evoked awe and respect” was not related to affect, as it correlated neither with hedonic nor eudaimonic feelings. These findings add more fine-grained knowledge about where and when hedonic and eudaimonic feelings occur during wilderness adventure. As there are similarities and differences between one group during forest-stormy and another group during plateau-cold, there is much more to understand than preference into different landscapes (Oishi et al., 2015). Also, as all students were presented with a new landscape, the bonding effect based on former experiences (Knez and Eliasson, 2017) could not explain the connection between place and well-being. In neither of the groups, students had been in this particular environment before. Rather, the finding that the sublime dimension has both context dependent and general features informs how to understand awareness and how to pay attention (Bergmann and Eaton, 2011) to this complex and powerful phenomenon.

### Affective, Experiential, and Philosophical Aspects Associated With “Feeling at Home in Nature”

The item that has the strongest affective relevance is the item “I felt at home in nature.” This was felt more intense during plateau-cold than during forest-stormy, with very high correlations during plateau-cold on both hedonic and eudaimonic feelings, while only hedonic feelings in forest-stormy. Interestingly, feeling at home in nature, as a theoretically strong argument for aesthetic nature experience, turned out to be the most affective item in the plateau-cold sample. Students were emotionally activated when they recalled an experience that they felt at home in nature. Informing strong affective aesthetic nature experiences, post measures of well-being correlated strongly with this item. During plateau-cold conditions, feeling at home correlated strongly with satisfaction with life, but this was not found during forest-stormy. On the other hand, personal growth was correlated with feeling at home in nature during both adventures. The finding supports that life satisfaction and personal growth needs to be understood separately as different facets of well-being (Vittersø, 2016). Moreover, it is informative that both expeditions related personal growth to feeling at home in nature, but life satisfaction was only related to feeling at home in nature during plateau-cold. Feeling at home in nature was felt affectively different during forest-stormy and plateau-cold. These are differences on group level, informing about contextual differences and identifying core characteristics across the groups. Nevertheless, the strong finding of feeling at home in nature as the most important feature, as it is felt strongest and alone predicted personal growth, leads to the connection of “Beheimatung” and “atmosphere” (Bergmann, 2011), which carries an awareness of who we are and how we are interconnected with nature, at least as an intense feeling and as an expression of personal growth.

The thematic analysis of narratives corresponding to “felt at home in nature” displays synthetic dimension relevant for our discussion. We recognize how the notion of “feeling at home in nature” and the interconnectedness in nature corresponds to descriptions of a certain moment with focus on sensory experiences, certain moments of appreciation of beauty, self-reflection, wonder, and situations focusing positive emotions (and even moments with combinations of these dimensions).

First, situations of feeling at home in nature take place when there is a certain moment with focus on sensory experiences. Revisiting the role of attention, feeling at home in nature includes narratives that follow some meaningful patterns in both samples, patterns that include attentional dynamics from the orienting and alerting network. Situations of feeling at home include perceptions of wilderness, such as contrasts of darkness/light and other visual sensory experiences as well as tactile ones, exemplified by sudden sights of stars, moon or northern lights, the warmth from the camp fire or the silence in forest. These examples of focused experience relate to the orienting network and can be explained as “soft fascination” (Kaplan and Kaplan, 1995).

One other synthetic dimension is appreciation of beauty. This dimension relates to appreciation of certain qualities, such as certain trees or landscapes, or the perception inside the snow cave, such as “insanely nice and an aha-experience.” Parsons (2008) says our experience of beauty is, bottom line, about our love to something. Talking about beauty in nature, he says, is about how people have strong feelings of love and attachment to certain places and things. Thus, the experience of beauty in nature is about much more than a disinterested contemplation.

Feeling at home strongly relates to positive emotions, with correlations to both hedonic and eudaimonic feelings. Three of the narratives associated directly to positive emotions, where two of them describe situations of movement in hedonic interpretations, like downhill skiing, walking and singing, and also enjoying breakfast. It is likely that eudaimonic feelings associated with feeling at home in nature thus include an active interpretation of a sensory experience, identified in the other synthetic dimensions, but there is also possible that this dimension is not fully understood in this explorative study. However, the strong connection between positive emotions and feeling at home in nature is observed. Positive emotions build our action repertoire and build resources to see the world in a more complex manner (Fredrickson, 2004). This could be a two-way process of (i) positive emotions empowering the feeling of being at home in nature and (ii) feeling at home, as a safe or inspiring moment, causing positive emotions, which in both cases promote our well-being. Feeling at home, as the strongest positive emotion, relates to Kaplan and Kaplan (1995, p. 193) interpretation of compatibility in finding a special resonance between natural environment and human inclinations. This resonance includes being away from civilization, living with less effort, supporting psychological well-being, but wilderness experience also leads to “a sense of awe and wonder and, at the same time, relatedness” (Kaplan and Kaplan, 1995, p. 194).

Feeling at home is also experienced when (i) a moment of self-reflection takes place, (ii) the experience of wonder is articulated and (iii) gaining insight of relation to nature. These dimensions are reflexive and philosophical by nature and could be related to each other. Description of self-reflection and gaining insight of relation to nature corresponds to the latter dimension of wonder, as evoking reflection on what we perceive and experience (e.g., being alone in the forest). The former dimension of wonder, evoked by something in nature, corresponds to the synthetic dimension of wonder which includes the experience of surprise and fascination of nature. Thus, in this context wonder, which is an active approach using the alerting network, takes place in in a fascination of trees, or experience a bird flying up. These synthetic dimensions correspond to self-reflection as one of the central components in wonder (Sæther, 2017), while sensory experiences and positive emotions, that also could relate to wonder, display as separate dimensions. Interestingly, in looking for experiences that correspond to feeling at home in nature, these wonder-experiences seem to be of the same characters in the two different contexts, but they had higher frequencies during plateau-cold.

Wonder spans over a wide range of meanings and is indubitably a complex phenomenon. Wonder can at least be distinguished in two ways: as something in nature which evokes a feeling of wonder and something motivating humans for self-reflection and further search for insight (Sæther, 2017). The former dimension of wonder has some similarities with the experience of beauty and the sublime. Experiences of beauty and sublime can take place as something striking you surprisingly from “the outside.” On the other hand, the latter experience of wonder takes place as a kind of reflection on what we perceive. Deane-Drummond (2009, p. 128) says: “… wonder is an even broader term than beauty and could be said to be prior to its recognition.” The comprehensiveness of wonder, broadly understood, is articulated by Schindler (2013, p. 163), describing wonder as “… a final state, as that-than-which-nothing-further-ought-to-be-sought.”

We also recognize complex experiences as we find combinations of the previous synthetic dimensions, such as both sudden feelings *and* reflection, which underscores that we cannot differentiate too categorically between the different dimensions. Even within the different dimensions, we find complex phenomena, such as wonder. Although we have to navigate carefully in such complex experiences in the wilderness, we will address one line of thought which displays the ethical relevance for feeling at home in nature (which include aesthetic experiences in nature, wonder and awareness). According to Robert Fuller (2012), wonder has some similarities with awe as experience. Awe transforms us toward a reorientation of our lives, goals, and values, he says. Further, awe evokes a feeling in us for being part of a larger whole. For Fuller, both wonder and awe are caused by novel and unexpected stimuli, challenging our given conceptual categories.

In light of the experiences of beauty and the sublime (awe) in wilderness, wonder as an experience can be understood as taking place together with, and in the extension of, beauty and awe (Sæther, 2019). When one experiences the beauty and the sublime in nature, this experience might evoke wonder (Ledley, 2009). Matravers (2012) characterizes this type of wonder as a reflective state. For Matravers, the experience of beauty and the sublime in nature evokes a first-order non-cognitive state, and the resulting feeling can be described as astonishment. Thus, wonder is about a duration of awareness, and corresponds to our theoretically unpacking of awareness, feeling at home, and how to pay attention to our surroundings – in Bergmann’s terms “living-in-particular-surroundings.” Therefore, our analysis of narratives informs about complex relationships when feeling at home in nature, where sudden feelings have hedonic and eudaimonic variation depending on context, and reflection seem to rise across many of the wilderness situations. Here, we recognize a trajectory from aesthetic experiences, through wonder, toward a potential ethical awareness. Such a trajectory corresponds to Bergmann’s notion of aesth/ethics, including his reflections on inhabitation and “Beheimatung.”

Feeling at home is also touched upon by García-Rivera (2009). According to him, in light of our environmental crisis, we need to address the question of being at home in the cosmos. For García-Rivera, an emphasis on “place” helps us to understand what this home or connectedness to nature might take shape, which corresponds to Bergmann’s notion of inhabitation. The narratives of feeling at home in the wilderness adventure identify several important places where feeling connected to nature are expressed directly, such as when being alone, looking at the stars or skiing in the forest. García-Rivera says place expresses both an inner as well as an outer dimension, and the experiences of such a place – in our context the experience of feeling at home in nature – is about an intimate immensity actual in space and time. Thus, aesthetic experiences of nature open for experiences of feeling at home which addresses deeper emotions involving belonging, interconnectedness. A recognition of such complex experiences might evoke ethical awareness.

### Strengths and Limitations

The empirical investigation of aesthetic wilderness experiences is based on experience reports from 47 individuals in a Norwegian study program. Strengths of the study design in understanding aesthetic wilderness experiences includes mixing quantitative measures as well as personal narratives as well as the examination of challenging environmental conditions. The utilization of mixed methods seems to be optimal in exploring deep connections in a complex field, as the integration of methods leads to more pinpointed knowledge: Quantitative measures inform intensity of aesthetic dimensions and feelings connected to these, while qualitative investigations gave feeling at home, identified as a quantitative finding, a much richer description and deeper interpretation (i.e., Løvoll, 2019).

One weakness with the current study was the hard work filling out the questionnaire in the evening, with cold fingers and in an uncomfortable condition. Consequently, we missed some participants, especially from the plateau-cold group, and also some narratives were very short. Nevertheless, we consider reliability as good, in comparison to other methods like using observational data or only retrospective data collection after adventures (Merriam and Tisdell, 2016).

Our two research contexts include one expedition with worse weather condition (forest-stormy) than the other (plateau-cold). As one of the contexts was under the tree line and the other above the tree line, landscape is one dimension of the context, and the other is the impact of weather, such as wind, temperature and snow. In the current analysis, we were not able to distinguish between other possible combinations of landscape and weather such as plateau-stormy or forest-cold.

To move forward, the study should be replicated with other groups and other contexts, including those outside Norway. Series of studies can produce empirical data for meta-analysis, calculating power for context dependent and context independent dimensions of the aesthetic wilderness experience. Moreover, more studies are needed to enable generalizing about the strong connection we found between feeling at home in nature and well-being.

### Implications

The findings have implications for theoretical and practical didactical reasons. In understanding the role of environment for aesthetic experiences, it is important to pay attention to wilderness, as the non-human-built environment offers a unique understanding of how aesthetic experiences appear and how those experiences connect to feelings and well-being. In this way, the knowledge of aesthetic wilderness experiences can offer a reference knowledge enabling comparison to aesthetic experiences within arts and built environments.

The finding that “feeling at home” is a very informative aesthetic dimension in being positively felt as well as important for well-being. This relates to belonging in a much wider sense, which also theoretically connects aesthetics to ethics. For the outdoor leader, there is a potential in identifying, dwelling, and cultivating what it is like to feel at home in nature, as this aesthetic dimension has individual importance, as well as a potential for ethical reflection. When considering learning outcomes in outdoor events or adventures, this dimension should not be under communicated or overlooked. In a contemporary context, where the UN’s climate report, and other initiatives, strongly call for human action in the face of global climate change, the connection between aesthetics and ethics is an important aspect in identifying, savoring and cultivating what “feeling at home in nature” actually means, in a global context, especially with an understanding of the role of wonder. Also, in a practical didactical context, the findings give impetus to focus on aesthetic wilderness experiences, as they provide strong emotional experiences as well as importance for well-being.

## Conclusion

This article has explored three complementary questions that relate aesthetic experiences and well-being within the wilderness environment. First, main dimensions of beauty and the sublime were identified as core features in situational wilderness experiences. The classical notion of beauty was more important in the plateau-cold condition.

Second, we found that “beauty in nature evokes wonder” was hedonic in forest-stormy and eudaimonic in plateau-cold. The item “felt at home in nature” had very high correlation on both hedonic and eudaimonic feelings during plateau-cold. In forest-stormy, this item only correlated with hedonic feelings. Hence, we find aspects of this feature to be context dependent. Feeling at home in nature correlated with both satisfaction with life and personal growth during plateau-cold while only with personal growth during forest-stormy.

Third, when exploring feeling at home in nature as an intense experience, six synthetic dimensions were identified through the narratives. These six dimensions relate to theories of wonder in different ways. To feel at home in nature can be understood as a non-intentional experience but also as an intentional experience when being self-aware. Our findings contribute to a more refined and systematic understanding of the characteristics of how wilderness experiences create aesthetic experiences and well-being. We accentuate that aesthetic experiences generate feelings of nature as our home and, further, creates an ethical awareness of the value of wilderness.

## Data Availability Statement

The datasets generated for this study are available on request to the corresponding author.

## Ethics Statement

Ethical approval was not provided for this study on human participants, because there was no person identifiable information in the collection of data. Details about the process is described in the Human Subjects Review section of the article. Written informed consent for participation was not required for this study in accordance with the national legislation and the institutional requirements.

## Author Contributions

All authors listed have made a substantial, direct and intellectual contribution to the work, and approved it for publication.

## Conflict of Interest

The authors declare that the research was conducted in the absence of any commercial or financial relationships that could be construed as a potential conflict of interest.
