# Supplementary material for: Feeling at Home in the Wilderness: Environmental Conditions, Well-Being and Aesthetic Experience
Source: Front Psychol. 2020 Mar 13;11:402. doi: 10.3389/fpsyg.2020.00402 (PMC7082929; doi:10.3389/fpsyg.2020.00402)
Supplement: Supplementary file 1 [file Data_Sheet_1.PDF]

Appendix I. *Thematic Analysis of Narratives Corresponding to "Felt at Home in Nature" = 7 on the Likert Scale*

Year	Narratives	Sub-themes (event)	Main themes (context)	Synthetic dimensions
2017	Full moon and pieces of starry sky<br>Beautiful view. We sat in front of the fire and worked in the camp with this view	Moon, light, communion	Contrast darkness / light	Description of a certain moment with focus on sense experiences
2018	When all the lights from the headlamp were extinguished and the stars appeared	Stars. Light		
	Look at the stars with a friend while we melted snow in 20 minus	Stars. Light. Cold		
	Sunset	Sun. Light. Change		
	The feeling of sitting around the fire and feeling the heat of the flames as I look up at a starry sky	Stars. Fire. Light		
	Saw the northern lights	Aurora Borealis. Light		
2017	Woke up first in the camp, out alone and brought with me the sun's first rays through the birch forest and the morning sun	Sun. Light. Alone		
	Come through the pine forest and feel the sun warming in the face and the sound of the wind whizzing in the trees	Sun warming, sound, forest	Sounds of wilderness	
	The wind, the ability to create silence through sound	Wind, sound, silence		
	Went through the winter forest (skiing) after the night's big snowfall. Silence and sense of presence	Forest, fresh snow, silence		
2018	Moving through the woods that seemed untouched	Moving. Forest. Untouched	Visual sense of wilderness while moving	
	To go uphill on the ski, while it glitters thousands of stars in the snow	Moving. Glittering in the snow. Light		
	The terrain we walked in: wants to ski all the "pillows" in the terrain	Moving, terrain, snow qualities	Tactile snow qualities while moving	
	Sleeping in snow cave	Snow cave	Snow experience	
	Freeze. Froze mostly all day. Intentionally. Did not change clothes.	Freezing intentionally	Testing clothes	
2017	The feeling of being alone in the pine forest	Being alone, forest	Forest reflection /Alone	Description of self-reflection
	Met on some fresh animal tracks	Animal tracks	Forest, surprise	Description of wonder
2018	Look at animal tracks in the forest			

	Saw many animal tracks			
	Ice-covered bench	Ice fascination	Fascination of details, Surprise	
	Big bird (Orrhane) that flew up	Bird		
2017	Snow cave. How amazing is it that you can build something so nice, cozy and warm by snow? Totally insanely nice and an aha experience	Snow cave	New snow experience	Description of a certain moment of appreciation of beauty
	We walked past a lovely old pine tree. The branches and trunk twisted around in a stylish way	Tree, forest	Fascination and aesthetic judgement of details / Forest	
2018	There were many trees that fascinated me		Fascination of details	
	Hilly wilderness landscape	Landscape	Fascination of details	
	When we walked between pine and birch and sang "In the forest I am free"	Walking and singing. Forest	Enjoyment	Situations focusing positive emotions
	Downhillskiing	Movement	Pleasure	
	When I had time to enjoy breakfast with the morning sun in the middle of me	Warmth from sun. Light		
2017	It had become completely dark. Everyone had gone to bed. I was still out with a good cup of cocoa and watched as the fresh snow came free down, illuminated by headlamp	Fresh snow, alone, dark / light	Contrast darkness / light Snow Alone	Description of a certain moment with focus on sense experiences **and** Description of self-reflection
	Went compass on the mountain (above the tree line) in strong winds. Felt the forces of nature and proximity to the mountain	Wind. Forces of nature	Reflection Wind	
	Wednesday night was all very wet and there was a night of varying amounts of sleep. On Thursday morning there was no wind, stay and lovely dry weather and bonfire did the day	Change. Dry weather	Weather and comfort change	Description of a certain moment with focus on sense experiences **and** Positive emotions
2018	When the fire burned well, the coffee cup was filled and the stars appeared (smiley)	Warmth from fire. Light	Contrast darkness / light	
	To get out into the snow in an amazing weather. Perfect for hanging up your sleeping bag	Change. Sunny weather. Light	Weather and comfort change	
	Sit in the sunshade with bonfire	Warmth from fire. Light	Pleasure	
	Lie in the sun by the fire and be warm	Warmth from fire. Sun.		
	Walk through the woods of skiing with everything you need to survive on your back makes you feel strongly connected to nature	Connection to nature, moving, carrying backpack, forest	Reflection on relation to nature when moving	Description of a certain moment with focus on sense experiences **and** Insight of relation to nature
	When I walked and pissed before sleep. Admired the view. The starry sky and that nature meets people	Stars. Light. Connection to nature	Contrast darkness / light. Reflection on relation while moving	

	Went a trip alone from the camp in the sunset up to hill, away from the others. Where the silence took over gazed over pointy mountains to the east. The color was glowingly warm and incredibly beautiful and used all the attention it needed to return to everyday life in camp. Also got the first day with toes without feelings.	Sun. Alone. Silence. Beauty. Comfort	Contrast darkness / light. Sounds of wilderness Alone	Description of a certain moment with focus on sense experiences **and** Description of self-reflection **and** Description of a certain moment of appreciation of beauty
